# High Serum β-Lactams Specific/Total IgE Ratio Is Associated with Immediate Reactions to β-Lactams Antibiotics

**DOI:** 10.1371/journal.pone.0121857

**Published:** 2015-04-16

**Authors:** Alessandra Vultaggio, Gianni Virgili, Francesco Gaeta, Antonino Romano, Enrico Maggi, Andrea Matucci

**Affiliations:** 1 Department of Biomedicine, Immunoallergology Unit, Policlinico di Careggi, Florence, Italy; 2 Department of Specialised Surgical Sciences, University of Florence, Florence, Italy; 3 Allergy Unit, Complesso integrato Columbus, Rome, Italy; 4 Center for Research, Transfer and High Education DENOTHE, University of Florence, Florence, Italy; Vrije Universiteit Brussel, BELGIUM

## Abstract

Total serum IgE result from the combination of specific and non-specific pools. High specific/total IgE ratio may reflect high level of allergen-specific IgE on mast cells. No data regarding its applications to drug allergies is available. One hundred seventy-one patients with a history of immediate reactions to β-lactams, confirmed by positive skin testing, and 122 control subjects tolerant to β-lactams, were studied. CAP System was used for the detection of serum total and specific IgE antibodies. The specific/total IgE ratio was tested for diagnostic accuracy compared with conventional criteria. We finally performed a simulation study to expand our investigation of the performance of the specific/total IgE ratio index in a scenario in which the new CAP detection threshold is lowered further. Specific/total IgE ratio values ≥0.002 were observed more frequently in reactive than in controls. Seventy-four of 80 subjects with values ≥0.002 were allergic to β-lactams, yielding a positive predictive value of 92.5%. The application of specific/total IgE ratio significantly improves the positive likelihood ratio and the overall diagnostic performance. In addition, we showed the capability of this new criterion to identify true reactive patients even among subjects with high levels of total IgE (>200 kU/L). Significant increase in both receiver operator characteristic (ROC) curve and sensitivity were observed in imputed case of the simulation study. The β-lactams-specific/total IgE ratio may be an additional index compared to the common criterion of positivity to a single hapten in the allergological work-up of patients with β-lactams immediate adverse reactions.

## Introduction

β-lactams are a frequent cause of adverse drug reactions (ADR) whose diagnosis is based on history, clinical examination, skin testing and demonstration of serum specific IgE antibodies [1]. When a mismatch between the history and the result of the primary diagnostic tools appears, the pathogenic role of a suspected culprit drug can be confirmed by provocation test. The detection of serum drug-specific IgE is relevant due to its safety and easiness, even though its sensitivity is low [2]. Moreover, the result of the test, and particularly its specificity, is largely affected by some additional factors such as serum total IgE levels [3].

Serum total IgE levels are not usually assessed since they are not considered useful in the clinical diagnosis of allergy and thus the ratio between specific and total IgE is almost never computed [4]. It has been reported that the percentage of allergen specific IgE is approximately 25% of total IgE, at least in respiratory allergy [5], and this proportion is usually much lower for β-lactam allergy. In fact, serum total IgE antibodies result from the combination of specific and non specific pools and variations in total IgE levels mainly reflect changes in specific IgE amounts [6]. It is well known that in type I hypersensitivity reactions mast cells and basophils activation is induced when a threshold number of allergen-specific IgE-charged FcεRI on their surface is cross-linked and aggregated by allergens [7]. Thus we can speculate that elevated specific/total IgE ratio might reflect high level of allergen-specific IgE on the mast cells and basophils surface and, thus, the increased probability of cell activation. In fact, we could hypothesise that in presence of high ratio values between sIgE and total IgE, the probability to have two next β-lactam-specific IgE on the basophil/mast cell surface membrane is higher, thus leading to an easier cross-linking of the FcεRI with following cells activation.

This ratio has been already used in allergological literature to predict the response to allergen-specific immunotherapy and to predict and prevent pediatric allergy [6,8]. However, no data regarding its application in drug allergy are available. Our study is aimed to evaluate the usefulness of serum β-lactam specific/total IgE ratio in improving the performance characteristics of current serological allergen (hapten)-specific IgE antibody assay. We also conducted a simulation study, based on missing imputation, to explore the potential for increasing diagnostic accuracy if the detection threshold of β-lactam specific IgEs could be lowered, allowing to investigate positive patients that cannot be currently identified.

## Methods

### Subjects

We enrolled a total of 171 β-lactams allergic patients (ADR+) who were referred to our attention for a recent immediate ADR to β-lactams. The positive diagnosis of β-lactams allergy was confirmed by skin testing positivity. Evaluation of atopic phenotype was based on history, clinical symptoms and positive skin prick test (SPT) for common allergens. Thirty-nine patients (39.2%) were atopic as defined by at least one sensitization to inhalant allergens and by the presence of related symptoms, and amoxicillin was the most frequent (88.5%) culprit drug. Most of them experienced urticaria (57.3%) or urticaria plus angioedema (7%). Anaphylaxis was recorded in 61 out of 171 patients (35.6%). A negative control group (ADR- patients) was also enrolled comprising 122 subjects (77 atopic and 45 non atopic) who reported tolerance to β-lactams administration in the last year and displayed negative skin testing. Written informed consent was obtained from all patients. This study exempted from institutional review board approval (Prot. 23.455-302/2009). Table 1 summarizes key characteristics of ADR+ and ADR- patients.

**Table 1:** Clinical characteristics of analyzed patients.

	ADR+	ADR-
Number (M/F)	171 (65/106)	122 (58–64)
Age (ys, range)	18–71	18–62
Allergic sensitization * (%)	67 (39.2)	77 (63.1)
Total serum IgE	554 ± 139	782 ± 127
Symptoms (%)		
Anaphylaxis°	61 (35.6)	-
Urticaria/Angioedema	12 (7)	-
Urticaria	98 (57.3)	-
Culprit drug (%)		
Amoxicillin	147 (85.9)	-
Ampicillin	13 (7.6)	-
Benzylpenicillin	6 (3.5)	-
Piperacillin	3 (1.8)	-
Bacampycillin	2 (1.2)	-
Positive skin test results (%)		
Penicilloyl-polylysine	14 (8.2)	-
Minor determinant mixture	36 (21)	-
Benzylpenicillin	49 (28.6)	-
Ampicillin	104 (60.8)	-
Amoxicillin	125 (73)	-
Positive specific IgE assay results (%)		
Penicilloyl G	59 (34.5)	-
Penicilloyl V	76 (44.4)	-
Ampicilloyl	97 (56.7)	-
Amoxicilloyl	68 (39.7)	-
Time delay (days)	89.1 ± 10.1	-

* confirmed by a positive skin testing to inhalant and/or food allergens. Total serum IgE were detected as described in Methods, values are reported as mean ± SE.

° patients who displayed a severe reaction affecting at least two organs with bronchospasm and/or hypotension.

### Skin testing

Skin testing was carried out as previously described with 0.02 ml of solutions [2]. The reagents were penicilloyl-polylysine (PPL, 5x10^-5^ M, Diater Laboratories, Spain), minor determinant mixture (MDM, 2x10^-2^ M, Diater Laboratories, Spain), amoxicillin (AMX, 20 mg/ml, GlaxoSmithKline, France) and ampicillin (AMP, 20 mg/ml, Pfizer, Italia). In both prick and intradermal testing a minimum wheal area of 3 mm diameter or an increase of area >3 mm was considered positive compared to a negative response to the saline control.

### Detection of serum specific and total IgE antibody

CAP System FEIA (Phadia, Uppsala, Sweden) based on ImmunoCAP technology has been used for the detection of both β-lactam-specific and total serum IgE antibodies according to the manufacturer’s instructions. All results were expressed in kU/l. Sera obtained from all patients were analyzed for IgE towards the hapten c1 (penicilloyl G), c2 (penicilloyl V), c5 (ampicilloyl), c6 (amoxicilloyl). Serum samples were considered positive when one or more hapten positivities occurred (detection limit of 0.10 kUA/l). Sera with a concentration of specific IgE antibodies below the detection limit were assigned an arbitrary value of 0.0 kUA/l. The specific/total IgE ratio was calculated making the sum of the measurements of β-lactam-specific IgE antibodies divided by total IgE.

### Statistical analysis

Receiver Operator Characteristic (ROC) curve was used to plot sensitivity against 1—specificity and compute the Area Under the Curve (AUC) using the percentile value method suggested by Janes and Pepe [9]. Sensitivity at 95% specificity was computed for the ratio as well as the conventional diagnostic criteria. Likelihood ratios (LR) were compared using formulas suggested by Hayen et al [10], and absolute differences in sensitivity or specificity were compared using negative binomial regression adjusting for within-patient correlation using Huber-White variance estimator. The Net Reclassification Improvement was used to calculate the difference in the number of correctly classified patients using a new criterion as an addition to a conventional one [11]. All calculations were done using Stata 13.0 software (StataCorp, College Station, Texas).

### Simulation study

In the simulation study, we wished to expand our investigation of the performance of the specific/total IgE ratio index in a scenario in which the new CAP detection threshold is lowered further. Specifically, we assumed that immeasurable specific IgEs could in fact be a mixture of cases in whom specific IgEs were truly absent, as compared to others in whom they were simply undetected since they were below CAP detection threshold. Therefore, we assessed the new diagnostic performance of CAP after imputing the specific IgE and total IgE joint distributions for cases in which specific IgEs were immeasurable.

As a first step we used Spearman correlation to assess whether the sum of specific IgEs correlated with total IgEs, separately by reactive and non-reactive patients. We plotted the sum of β-lactam vs. total IgEs, with both axes on a log scale, mainly because this transformation tends to normalize such skewed distributions and, secondarily, enables to plot the ratio index values as a straight line, given that the ratio of two variables equals a linear difference in their logs.

We performed multiple imputation using chained equations methods to impute missing, i.e. immeasurable, levels of the sum of β-lactam IgEs [12]. We simulated 20 datasets of “missing”β-lactam IgEs based on total IgE value, both on the log scale. Because we wanted to extrapolate the joint distribution of specific IgE sum and total IgEs to fill in missing values while preserving the observed performance of the ratio index, missing imputation included a dichotomous variable coding for ratio index positive and negative patients, using the cut-off identified at 95% specificity, and was done separately for reactive and non-reactive patients. The imputation was based on linear regression and thus tended to impute immeasurable values of β-lactam IgE sum assuming a multivariate normal distribution of the imputed variable and total IgEs, the latter fully observed. Using log IgE values, only positive values, although very small ones, can be generated. This approximation of nil values to extremely low values was intended and acceptable when the aim is to estimate the diagnostic ability of the ratio index, as recalculated using imputed data.

## Results

### Diagnostic performance of specific/total IgE ratio criterion

**Fig 1:**
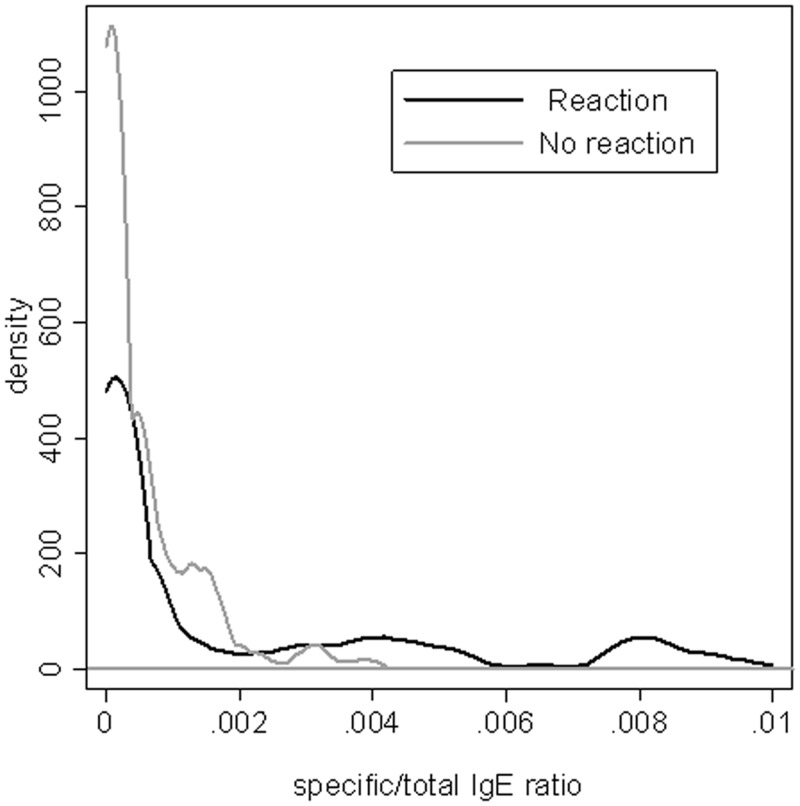
Smoothed distribution of the specific/total IgE ratio in reactive and non-reactive patients.

**Fig 2:**
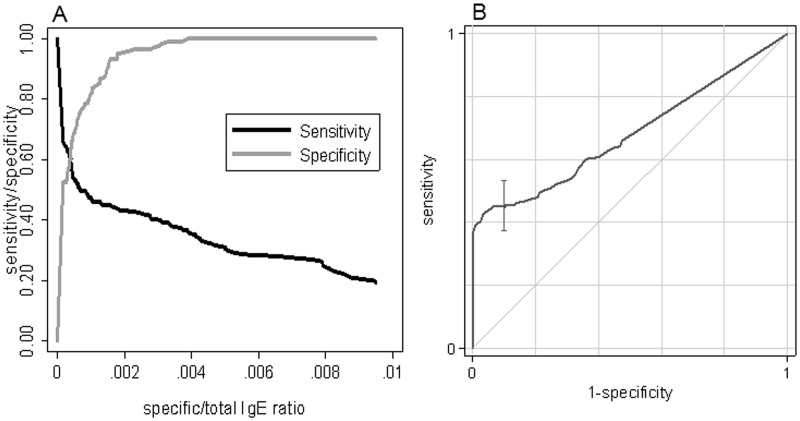
Diagnostic accuracy of the specific/total IgE ratio for detecting reactive patients. Accuracy is presented both as sensitivity and specificity plot at increasing ratio levels (2A: left) and as a ROC curve (2B: right). The uncertainty of the specificity at 90% specificity is also shown as a vertical bar in the ROC curve.

We evaluated the distribution of specific/total IgE ratio values in patients with a confirmed history of immediate adverse reaction to β-lactams, compared to negative control subjects. As shown in Fig 1, there is a substantial overlapping of the distribution of the values up to ratio of 0.002, whereas higher ratio values were observed in reactive subjects. A specific/total IgE ratio value of 0.002 was found to be the cut-off at 95% specificity. We then analyzed sensitivity and specificity of the increasing levels of specific/total IgE ratio to predict allergic reactions by using ROC curves (Fig 2A). Using the ratio index in the observed dataset, the AUC was 0.68 (95% CI: 0.62–0.74), which is a modest overall diagnostic performance taking into account that a good performance is when a AUC is at least 0.80. However, the curve was markedly asymmetric, showing a better performance at high specificity than at high sensitivity (Fig 2B). In fact, the diagnostic performance at 95% specificity (ratio cut-off 0.002) was a sensitivity of 43.3%, and 190 out of 293 subjects (64.8) were correctly classified (true positive and true negative). As seen in Fig 2A, specificity was almost maximized at specific/total IgE ratio ≥0.002. These values are more useful for ruling in disease using positive LR (LR+:8.8, 95%CI: 4.0–19.6) rather than excluding it using negative LR (LR-:0.60, 95%CI: 0.52–0.68). Seventy-four out of 80 subjects with specific/total IgE ratio values of 0.002, or more, were allergic to β-lactams, yielding a positive predictive value of 92.5% in this sample. In order to assess the impact of each hapten on ratio diagnostic performance, we excluded it from ratio computation and compared AUCs. With reference to the ratio including all haptens (AUC: 0.68), excluding PenG and Amp did not alter results (AUC: 0.67 in both cases, p = 0.472 and p = 0.487, respectively), whereas excluding PenV and AmoX minimally but significantly decreased accuracy (AUC: 0.66, p = 0.042 and AUC: 0.65, p = 0.019, respectively). Such modest differences do not suggest a differential role of any hapten in ratio performance.

### Comparison of different diagnostic criteria

In clinical practice CAP for β-lactams is considered positive when specific IgE antibodies were present for at least one hapten. It has been previously showed that the diagnostic accuracy of CAP assay is greatly influenced by total serum IgE levels [3]. The diagnostic performance of a CAP test for β-lactams (cut-off positivity >0.10 kUA/l for at least one hapten) is maximized for patients with low to moderate total IgE levels (<200 kU/l) [3]. Table 2 summarizes the diagnostic performance, including diagnostic odd ratio (DOR), for different criteria of positivity. The application of specific/total IgE ratio >0.002 is better than conventional positivity, since it significantly improves the LR+ (*P* < .001). In fact, as reported in Table 2, the overall diagnostic performance expressed by the DOR value is higher using the proposed specific/total IgE ratio cut-off rather than the standard criterion.

**Table 2:** Diagnostic performance of different criteria.

	Criteria of CAP positivity		
	1 positive hapten	1 positive hapten and total IgE<200*	Specific/Total IgE ratio >0.0022
Sensitivity	0.66 (0.59–0.73)	0.34 (0.27–0.42)	0.43 (0.36–0.51)
Specificity	0.52 (0.42–0.61)	0.98 (0.94–1.0)	0.95 (0.90–0.98)
Positive predictive value	0.66 (0.58–0.73)	0.97 (0.89–1.0)	0.93 (0.84–0.97)
Negative predictive value	0.52 (0.43–0.61)	0.52 (0.45–0.58)	0.55 (0.48–0.61)
Positive likelihood ratio	1.4 (1.1–1.7)	20.7 (5.2–83)	8.8 (4–19.6)
Negative likelihood ratio	0.66 (0.50–0.86)	0.67 (0.60–0.75)	0.6 (0.52–0.68)
Diagnostic odds ratio	2.1 (1.3–3.3)	30.8 (8.1-NE)	14.7 (6.3–34.5)

*kU/l, NE: not estimable.

At the specific/total IgE ratio cut-off associated with 95% specificity in this study (0.002), a larger number of allergic subjects can be classified as positive (n = 74) compared to previous combined criteria of at least one hapten positivity in the presence of total IgE<200 kU/l (n = 58); this is a modest, but statistically significant increase in sensitivity from 35% to 45% (*P* < .001). The small difference in specificity (98% vs 96%), causes a large, but non statistically significant LR+ reduction, due to missing 2 vs. 6 reactive patients with the conventional vs. the ratio criterion.

As reported above, in our case series we identified 80 subjects with specific/total IgE ratio >0.002, of whom 74 (93%) resulted true positive. Twenty-one out of these 80 patients displayed high levels of total IgE (>200 kU/l). However, most of them (17/21; 81%) were really ADR+, thus showing the capability of this new criterion to identify true reactive patients even among subjects with high levels of total IgE. When specific/total IgE ratio >0.002 was applied to reactive patients with at least one hapten positivity and low levels of total IgE (<200 kU/l) (n = 58), we observed that all but one was correctly identified by the ratio criterion (57/58, 98%).

Overall, there was a gain in the number of correctly classified patients using the ratio criterion. The Net Reclassification Improvement achieved adding the ratio to the single apten positivity criterion was 20.6% (p = 0.003). That obtained adding the ratio to the combination of apten positivity and total IgE<200 was 6.7% (p = 0.022). The Net Reclassification Improvement allowed by the ratio index in patients with total IgE>200 kU/l was 22% over at least one hapten positivity (p = 0.005)

### Simulating RAST performance under improved β-lactam IgE detection threshold

**Fig 3:**
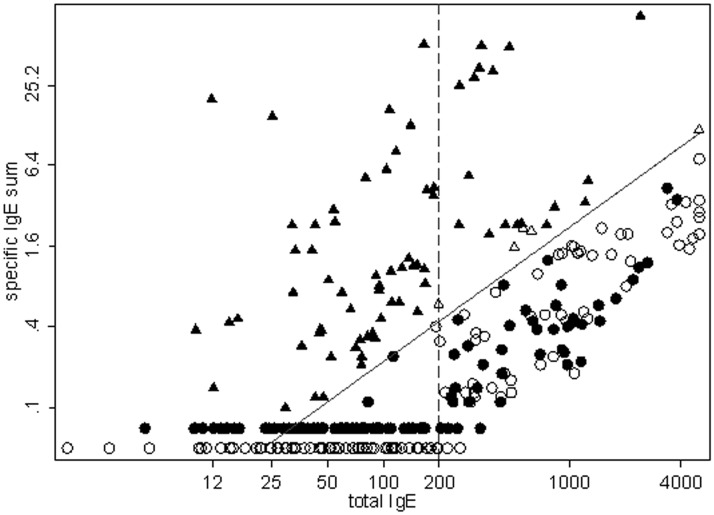
Scatterplot of the sum of β-lactams vs. total IgEs. Both axes are on the log scale, enabling to plot the ratio index values as a straight, oblique line, given that the ratio of two variables equals a linear difference in their logs. Black filled symbols are reactive patients and empty symbols are non-reactive subjects. Triangles are subjects with specific/total IgE ratio above 0.002 and circles are those below this level. Subjects with non-measurable sum of β-lactam IgEs are plotted in two lines parallel to the x axis and below the detection threshold of 0.10 kUA/l. It can be seen that the subjects above the ratio are nearly all reactive or true positives (black triangles), while a substantial amount of false positives (black circles) is found below this ratio threshold for subjects with total IgE above 200 kU/l.

**Fig 4:**
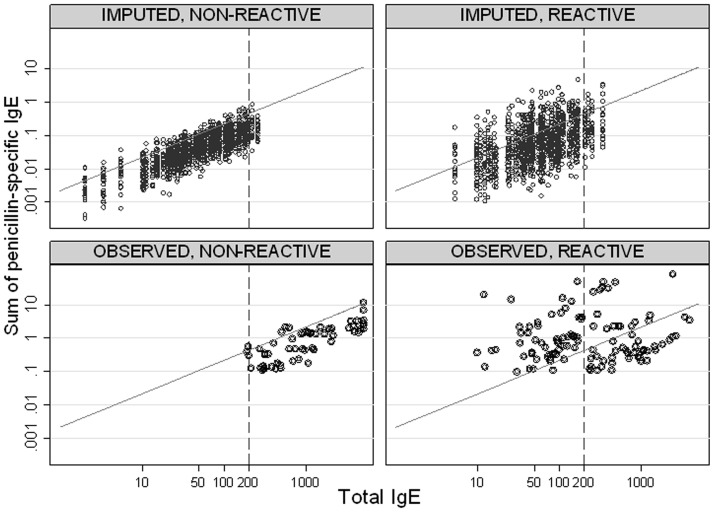
Scatterplot of the sum of β-lactam vs. total IgEs for observed and imputed values. Observed values (bottom row) and imputed values (upper row) as explained in the Methods. Observed and imputed data for non-reactive patients (left column) tend to line just below the ratio index threshold oblique line corresponding to 0.002 cut-off. See text for comments.

Using observed data, a very strong correlation was observed between total and specific IgEs for non-reactive patients (overall Spearman coefficient: 0.79, p<0.001) as compared to a modest correlation in reactive patients (Spearman coefficient: 0.44, p = 0.062). This high correlation among non-reactive observed patients can be appreciated in Figs 3 and 4, since the sum of specific IgEs tend to line just below the ratio index threshold oblique line corresponding to 0.002 cut-off, meaning that about 1/1000 total IgEs are consistently, but wrongly, measured as specific IgEs. Clearly, such a fraction is below current CAP detection threshold for total IgE values below 0.1 kU/l.

Specifically Fig 3 presents the classification of all subjects using the ratio cut-off of 0.002 or more (triangles) vs. below 0.002 (circles) together with the reactive status (reactive: solid black symbols; non-reactive: hollow white symbols), In this figure, results are presented according to the log of specific IgE sum (y-axes) vs. total IgE (x-axes), and their 0.002 ratio value is seen a straight, oblique line as explained in the Methods. For convenience, specific IgE values below 0.1 kU/l, i.e. immeasurable are displayed at fixed values, since the log of zero cannot be taken. It appears that all subjects above the ratio are reactive, but also that a substantial mixture of reactive and non-reactive patients is found below a ratio 0.002. Nil or immeasurable β-lactam IgE sum is almost only found below total IgE of 200 kU/l.

**Fig 5:**
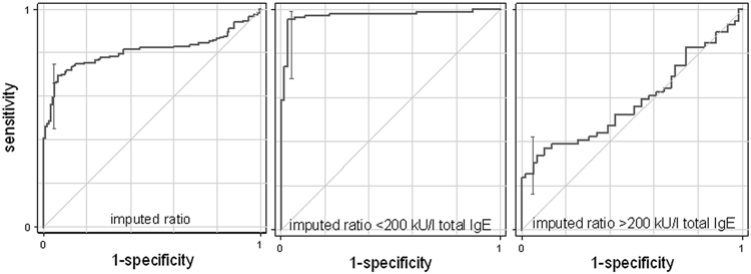
ROC curves analysis. ROC curves (with vertical line presenting the 95% confidence interval of sensitivity at 90% specificity) for imputed specific/total IgE ratio in the whole sample (left), as well as in subjects with total IgE values below (center) or above (right) total IgE values of 200 kU/l.

Fig 5 presents the improvement of the ROC curve after imputation compared to the observed ratio index (Fig 2B). In fact the AUC improves from 0.68 (0.62–0.74) to 0.81 (95%CI: 0.76–0.86) respectively. Similarly the sensitivity at 95% specificity from 0.44 (0.36–0.52) to 0.80 (0.67–0.92). Differences in both AUC and sensitivity were significant at p<0.001 level. The bottom part of Fig 5 shows ROC curves separately for patients below (left) and above (right) 200 kU/l. While the performance for patients with lower IgE values was almost perfect, that for patient with higher values was modest, with AUC of 0.95 (0.92–0.99) vs. 0.60 (0.47–0.68), respectively, which is consistent with most specific IgE values being imputed at low total IgE levels.

## Discussion

Our study shows that the ratio of the sum of β-lactam specific to total IgE is able to improve the diagnostic performance of CAP assay in the identification of reactive patients. In particular, our data suggest the clinical usefulness of the ratio at least in patients with serum total IgE>200 kU/l.

The elevated value of the specific/total IgE ratio observed in our β-lactams reactive patients is in accordance with the biological background of the mast cells/basophils IgE-induced activation. In fact, these effector cells are activated anytime a threshold number of hapten-specific IgE-charged FcεRI on them is cross-linked by hapten molecules [7]. The specific/total IgE ratio is of great importance since it may reflect a high level of allergen (hapten)-specific IgE (mast cells/basophils) which are the actors of type I hypersensitivity. An elevated specific/total IgE ratio may be associated with a high probability that β-lactam specific IgE on the mast cells are very close, whereas this is rare when the ratio is low. The density of FcεRI occupied by hapten-specific IgE on the surface of mast cells and basophils are related to the proportion of hapten-specific IgE and the concentration of total IgE. The sum of the IgE values that provides the numerator for the ratio, probably overestimates the true fraction of specific IgE. However in our study this bias is overcome by the application of the same criterion in both reactive and non reactive patients.

Compared to inhalant and food allergens, β-lactam-specific IgE antibodies are generally lower and the dilution effect of total IgE levels may have a major impact on effector cells response. In β-lactam unreactive patients the low concentration of the pathogenic IgE antibodies cannot reach the threshold level able to trigger mast cells. Considering these data, one may take into account the total IgE and should interpret specific IgE levels differently in patients with high or low total IgE levels.

The clinical significance of the ratio value is also suggested by the data showing the relationship between the size of the disease-relevant IgE fraction and total IgE, and the efficacy of anti-IgE (omalizumab) treatment [13]. This is also in agreement with a recent study showing that the serum specifc/total IgE ratio significantly correlates with the clinical response to allergen specific immunotherapy in pollen-reactive patients and in foods allergy [8,14]. The effect of total IgE serum levels on the diagnostic performance of the CAP system in order to identify β-lactam-reactive patients has been previously proposed [3]. We suggest that β-lactam specific/total IgE ratio may be a further diagnostic index to study subjects with suspected β-lactam allergy. In fact, an advantage of this index lies in the fact that it is a continuous measure and the balance between sensitivity and specificity can be set. This is important since CAP performance is better at high specificity rather than at high sensitivity, due to the highly variable values of both CAP results and total IgE in reactive patients. This leads to a substantial overlapping of reactive and nonreactive populations in the low range of values, specifically below the specific/total IgE ratio of about 0.002,.

Our simulation study shows that the misclassification of the reactive status in patients with immeasurable β-lactam IgEs, almost all of whom have total IgE values <200 kU/l, is a major limitation of the accuracy of CAP testing. The very high correlation of β-lactam and total IgEs in non-reactive patients suggests that the source for false negative findings is that CAP wrongly measures a fraction of total IgEs as β-lactam IgEs. This fraction is consistently seen as less than 2/1000, our ratio cut-off, in Fig 5. Thus, it would be less than 0.1 kU/l for patients with total IgE values <200 kU/l, making the index useless below this value. This is confirmed by our simulation study, which suggests potential for a great improvement of the ratio diagnostic performance were the detection threshold lowered. Further improvement of CAP testing could be only achieved if misclassification of β-lactam and total IgEs is avoided.

Our study aims primarily to compare different diagnostic expressions of CAP results to be used in future research in this field, and to estimate the diagnostic accuracy of the new index—the specific/total IgE ratio. However, some limitations to the interpretation of the data presented regarding the estimate of diagnostic accuracy, should be acknowledged. We used patients’ history of reaction combined to positive skin testing results as a reference, in order to create well-defined groups of reactive and non reactive subjects. Thus, one can maximize CAP diagnostic performance with the aim of exploring differences among diagnostic criteria. This case-control design overestimates diagnostic accuracy of a test as compared to its use in a series of consecutive cases [15,16]. A weakness of the data is that we did not use challenge test results as reference. However, we have to take into account that the provocation test with culprit drug may be dangerous, particularly in patients with drug-induced anaphylaxis (35.6% of our cases).

In conclusion, the IgE-based criterion proposed here should be used in studies investigating the diagnostic performance of CAP to detect β-lactams allergy. Future diagnostic accuracy studies should recruit subjects as a consecutive series, avoiding the exclusion of cases with uncertain β-lactams allergy status. In particular, the analysis of ratio in confounding cases, such as patients with suggestive history but negative skin testing, should give additional information on the applicability of our finding to real-life patients management. Large-scale multicenter studies are advisable to obtain the necessary statistical power and ensure the generalization of results. Finally, we suggest there would be great improvement in using the ratio criterion provided that technological development succeeds in lowering CAP detection threshold.

## Supporting Information

S1 Data (XLS)

## References

[1] Khan DA, Solensky R (2010) Drug allergy. J Allergy Clin Immunol 125:S126–37. 10.1016/j.jaci.2009.10.028 20176256

[2] Torres MJ, Blanca M, Fernandez J, Romano A, de Weck A, Aberer W, et al (2003) ENDA: EAACI Interest Group on Drug Hypersensitivity. Diagnosis of immediate allergic reactions to beta-lactam antibiotics. Allergy 58:961–72. 14510712 10.1034/j.1398-9995.2003.00280.x

[3] Vultaggio A, Matucci A, Virgili G, Rossi O, Filì L, Parronchi P, et al (2009) Influence of total serum IgE levels on the in vitro detection of β-lactams-specific IgE antibodies. Clin Exp Allergy 39:838–44. 10.1111/j.1365-2222.2009.03219.x 19400911

[4] Kerkhof M, Dubois AEJ, Postma DS, Schouten JP, Monchy JGR (2003) Role and interpretation of total serum IgE measurements in the diagnosis of allergic airway disease in adults. Allergy 58:905–11. 12911420 10.1034/j.1398-9995.2003.00230.x

[5] Jackola DR, Blumenthal MN, Rosenberg A (2004) Evidence for two independent distribution of serum immunoglobulin E in atopic families: cognate and non-cognate IgE. Human Immunol 65:20–30. 14700592 10.1016/j.humimm.2003.10.012

[6] Matricardi PM, Bockelbrink A, Grüber C, Niggemann B, Hamelmann E, Wahn U, et al (2009) Longitudinal trends of total and allergen-specific IgE throughout childhood. Allergy 64:1093–98. 10.1111/j.1398-9995.2009.02055.x 19630859

[7] Ishizaka T, Conrad D, Schulman ES, Sterk AR, Ko CG, Ishizaka K (1984) IgE-mediated triggering signals for mediator release for human mast cells and basophils. Fed Proc 43:2840–45. 6207053

[8] Di Lorenzo G, Mansueto P, Pacor ML, Rizzo M, Castello F, Martinelli N, et al (2009) Evaluation of serum s-IgE/total IgE ratio in predicting clinical response to allergen-specific immunotherapy. J Allergy Clin Immunol 123:1103–10. 10.1016/j.jaci.2009.02.012 19356792

[9] Janes H, Pepe MS (2008) Adjusting for covariates in studies of diagnostic, screening, or prognostic markers: An old concept in a new setting. Am J Epidemiol 168:89–97. 10.1093/aje/kwn099 18477651

[10] Hayen A, Macaskill P, Irwig L, Bossuyt P (2010) Appropriate statistical methods are required to assess diagnostic tests for replacement, add-on, and triage. J Clin Epidemiol 63:883–91. 10.1016/j.jclinepi.2009.08.024 20079607

[11] Pencina MJ, D'Agostino RB, Pencina KM, Janssens AC, Greenland P (2012) Interpreting incremental value of markers added to risk prediction models. Am J Epidemiol 176:473–81. 22875755 10.1093/aje/kws207 PMC3530349

[12] White IR, Royston P, Wood AM (2011) Multiple imputation using chained equations: Issues and guidance for practice. Statistics in Medicine 30: 377–99. 10.1002/sim.4067 21225900

[13] Johansson SG, Nopp A, Oman H, Ankerst J, Cardell LO, Grönneberg R, et al (2009) The size of the disease relevant IgE antibody fraction in relation to “total IgE” predicts the efficacy of anti-IgE (Xolair) treatment. Allergy 64:1472–77. 10.1111/j.1398-9995.2009.02051.x 19393000

[14] Federly TJ, Jones BL, Dai H, Dinakar C (2013) Interpretation of food specific immunoglobulin E levels in the context of total IgE. Ann Allergy Asthma Immunol 111:20–24. 10.1016/j.anai.2013.05.012 23806455

[15] Johansson SGO, Adedoyn J, van Hage M, Gronneber R, Noop A (2013) False-positive penicillin immunoassay: An unoticed common problem. J Allergy Clin Immunol 132:235–37. 10.1016/j.jaci.2012.11.017 23270810

[16] Rutjes AW, Reitsma JB, Vandenbroucke JP, Glas AS, Bossuyt PM (2005) Case-control and two-gate designs in diagnostic accuracy studies. Clin Chem 51:1335–41. 15961549 10.1373/clinchem.2005.048595

